# sORFs.org: a repository of small ORFs identified by ribosome profiling

**DOI:** 10.1093/nar/gkv1175

**Published:** 2015-11-02

**Authors:** Volodimir Olexiouk, Jeroen Crappé, Steven Verbruggen, Kenneth Verhegen, Lennart Martens, Gerben Menschaert

**Affiliations:** 1 Lab of Bioinformatics and Computational Genomics (BioBix), Department of Mathematical Modelling, Statistics and Bioinformatics, Faculty of Bioscience Engineering, Ghent University, 9000 Ghent, Belgium; 2 Department of Medical Protein Research, VIB, 9000 Ghent, Belgium; 3 Department of Biochemistry, Ghent University, 9000 Ghent, Belgium

## Abstract

With the advent of ribosome profiling, a next generation sequencing technique providing a “snap-shot’’ of translated mRNA in a cell, many short open reading frames (sORFs) with ribosomal activity were identified. Follow-up studies revealed the existence of functional peptides, so-called micropeptides, translated from these ‘sORFs’, indicating a new class of bio-active peptides. Over the last few years, several micropeptides exhibiting important cellular functions were discovered. However, ribosome occupancy does not necessarily imply an actual function of the translated peptide, leading to the development of various tools assessing the coding potential of sORFs. Here, we introduce sORFs.org (http://www.sorfs.org), a novel database for sORFs identified using ribosome profiling. Starting from ribosome profiling, sORFs.org identifies sORFs, incorporates state-of-the-art tools and metrics and stores results in a public database. Two query interfaces are provided, a default one enabling quick lookup of sORFs and a BioMart interface providing advanced query and export possibilities. At present, sORFs.org harbors 263 354 sORFs that demonstrate ribosome occupancy, originating from three different cell lines: HCT116 (human), E14_mESC (mouse) and S2 (fruit fly). sORFs.org aims to provide an extensive sORFs database accessible to researchers with limited bioinformatics knowledge, thus enabling easy integration into personal projects.

## INTRODUCTION

Small open reading frames (sORFs) can be defined as open reading frames smaller than or equal to 300 nucleotides (100 amino acids). These ‘sORFs’, while inherent to all genomes, were historically ignored in gene annotation studies, stating that these lack any coding potential (1). Mainly due to their small size they were thought to occur by chance, however, some longer sORFs resemble protein-coding ORFs and thus simplify their annotation. Exclusion of these sORFs has emerged during the development of different (gene prediction) tools in the field of bioinformatics/genomics/proteomics trying to reduce noise, imposed by technological limitations. For *in silico* prediction sORFs are excluded because these can easily occur by chance due to their small size. RNAseq driven transcriptomics is ignorant to ORF delineation and thus mainly focuses on the longest available ORF in the transcript sequence. As for MS-based proteomics studies, the small protein products are often lost in sample preparation steps and furthermore micropeptides are thought to be low abundant and can have tissue/time specific expression, further impeding their identification. The search for micropeptides, defined as translation products from sORFs, was nourished with the advent of ribosome profiling (2,3), a next generation sequencing technique. Ribosome profiling (RIBO-seq) recovers and subsequently sequences the ±30 nt RNA fragments captured within translating ribosomes. This technique differs from a regular RNA-seq setup, as a ‘snap-shot’ is provided of what is being translated in a cell, rather than what is expressed in a cell. In this context, it allows to detect translated sORFs, possibly encoding functional peptides or small proteins. Standard RNA sequencing techniques are unable to detect translated sORFs. Mass spectrometry is routinely used to detect and measure translation products. Although this technique is rapidly improving in sensitivity, detection of translating sORFs remains very difficult, making RIBO-seq (4) the preferred tool for sORF discovery. Also, RIBO-seq enables translation initiation site (TIS) detection through specific antibiotics treatment using harringtonine (HARR) or lactimidomycin (LTM). These drugs make that initiating ribosomes are stalled at the translation initiation site as opposed to the normal procedure where all translating ribosomes are obtained after cycloheximide (CHX) treatment. While RIBO-seq provides data on many putatively functional translated sORFs, ribosome occupancy does not automatically imply true coding and function at the peptide level. Consequently several tools/metrics have been published in order to assess the coding potential (i.e. the potential to encode functional peptides) of RIBO-seq/sORFs/micropeptide related data. Analytical methods measuring the coding potential can be either sequence based: multiple sequence alignment-based phylogenetic analysis, sequence variation or based on RIBO-seq: sequence similarity analysis ribosome protected fragment (RPF) length analysis, RPF reading frame analysis. Despite the onerous proteomic identification of micropeptides, it is still the best methodology to truly (at amino acid level) identify micropeptides. Since the advent of RIBO-seq, the biological functions of several micropeptides were unraveled. Toddler, for example, is an embryonic signal that promotes cell movement (5), Pri-peptides regulate various development steps across many insect species (6), Sarcolipin regulates muscle-based thermogenesis in mammals (7) and Myoregulin regulates Ca (2+) handling in muscle cells (8). These examples highlight the uprising importance of micropeptides (9–11). The creation of a public repository for sORFs, holding a growing number of RIBO-seq studies and providing information resulting from various tools and metrics, seems a necessity in aiding the necessary functional research in the micropeptide field. Here, we present www.sorfs.org, a comprehensive repository of sORFs identified by RIBO-seq, currently harboring 263 354 sORFs originating from three different species (human, mouse, fruit fly).

## MATERIALS AND METHODS

### Database development

The current sORF identification pipeline requires RIBO-seq data after both CHX-treatment, capturing elongating ribosomes, and HARR- or LTM-treatment, resulting in initiating ribosomes (12). The RIBO-seq sequence reads are first aligned using the STAR splice site aware mapper (13), as described by the PROTEO-FORMER pipeline (14). Reference genome indexes and gene annotation information are retrieved from the iGenomes repository (based on Ensembl annotation version 75, https://support.illumina.com/sequencing/sequencing_software/igenome.html) and are updated on every new release. A summary of parameters, mapping statistics as well as quality control files (FastQC (15)) can be found on the sorfs.org ‘data sets’ page. Secondly, the translation initiation sites are determined using criteria defined by Lee *et al*.(16). A full description of the TIS-calling implementation can be found in the PROTEOFORMER pipeline (14). Subsequently, sORFs are assembled starting from the detected TIS positions extending the sequence to the next stop codon situated 10–100 amino acids further upstream and in-frame relative to the TIS. Here, existing gene annotation information can optionally be taken into account (either or not splice-aware). Alongside the genomic positions a number of general sORF related characteristics are calculated. These include the mass of the resulting peptide, the mRNA and peptide sequence, a categorization based on the Ensembl mRNA annotation (5′ UTR, exonic, intronic, 3′ UTR, ncRNA or intergenic). For intergenic sORFs the distance to the nearest up- and downstream gene is calculated and for each 5′ UTR, exonic or intronic sORF the percentage of overlap with exonic regions is retrieved and a possible frameshift is determined relative to the overlapping Ensembl transcript. The RPF and RPF-fragments per kilobase of coding region per million aligning reads (RPKM) are computed as described in Ingolia *et al*. (2). A unique ID is provided to all identified sORFs, constructed from the corresponding cell line and an auto-incremental number as follows: [cell line]:[auto-incremental number]. All data are generated using in-house Perl (version 5.16.3) and Python (version 2.7.10) scripts and stored in a MySQL database (version 5.5.42). Currently sORFs.org holds three RIBO-seq data sets from three different cell-lines: HCT116 (human colon cancer cell line), E14_mESC (Mouse embryonic stem cells, 14 days old) and S2 (20–24 h old Drosophila melanogaster embryos). A detailed overview of the cell lines can be found at http:/www.sorfs.org/dataset_information. With every iGenomes update, data will be reprocessed and updated within the next month. New data sets are actively searched for and will be included if permitted by the owners, after a manual inspection of the data (quality control) and should be expected to be included within the next month. Same holds for data submitted by users.

**Figure 1.**
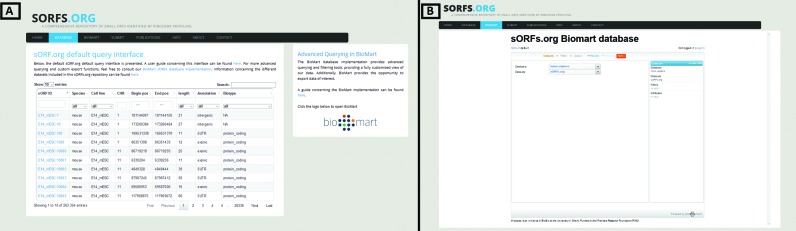
(**A**) sorfs.org default query interface. (**B**) sorfs.org BioMart query interface.

The sorfs.org web interface was build using the laravel PHP-framework (version 4.2), applying the model-view-controller (MVC) architectural paradigm. The web interface was developed using HTML, PHP, CSS, SQL and JavaScript. Two different query interfaces are provided to the user. The default query interface (see Figure 1A) provides real-time lookup of sORFs with limited query possibilities, excelling in the quick lookup of specific sORFs. Secondly a BioMart (17) (version 0.9.0) query interface (see Figure 1B) was developed enabling advanced query and export options. A comprehensive guide for both query interfaces is provided on sORFs.org.

### Coding potential assessment

#### Based on sequence conservation

Several algorithms are implemented providing coding evidence of the identified sORFs. A PhyloCSF conservation analysis (18) uses species-specific multiple alignment files from UCSC (19) in order to obtain a score representing the phylogenetic conservation of a sORF. PhyloCSF examines evolutionary signatures characteristic to alignments of conserved coding regions in order to determine whether a multi-species nucleotide sequence alignment is likely to represent a protein-coding region.

#### Based on ribosome profiling data

(i) The fragment length organization similarity score (FLOSS), described by Ingolia *et al*.(20), measures the magnitude of disagreement between the RPF-length distribution of Ensembl annotated protein coding sequences and the RPF-length distribution of a sORF. This fragment length metric enables to identify true ribosome footprints bioinformatically. Additionally a classification is formalized by defining a threshold FLOSS value. (ii) The ORFscore, a novel metric described by Bazinni *et al*.(21), quantifies the preference of RPFs to accumulate in the first frame of the coding sequence, as an indication for true coding sequences. The ORFscore, specifically designed for small ORFs, is calculated by counting RPFs in each frame and subsequently comparing this distribution to an equally sized uniform distribution using a modified chi-squared statistic. Only RPFs with length corresponding to the most abundant, in-frame RPF found in the Ensembl canonical protein coding transcripts, are used. For example if the annotated Ensembl CDS contains mostly 29-bp long footprints, only these 29 bp footprints will be used for the ORFscore analysis within this region.

#### Based on sequence variation

Sequence variation (i.e. mutations, insertions or deletions) associated with distinct phenotypes provides information on the function of that genomic/mRNA region. Associating sequence variation with sORFs provides evidence for functionally important sORFs. The Ensembl variation database (22,23) (including dbSNP, ClinVar, Cosmic …) is used as the source for sequence variation. Important to note: no filters were applied on these variation sources; caution is advised as some sources contain machine-annotated variations.

#### Based on sequence homology

Sequence similarity between sORFs and known proteins can discover false positives sORF annotations (e.g. a 5′ UTR sORF matching an unannotated protein isoform). The ‘Basic Local Alignment Search Tool protein’ (BLASTp) (24,25) was used to calculate AA-sequence similarity between sORFs and the Non-redundant (NR) protein sequence database (NCBI) (26). An expected value (E-value) of 10 holds as an upper threshold to define adequate similar sequences.

In order to provide some insight into various sORF attributes (TIS distribution, Ensembl annotation, PhyloCSF, FLOSS, variation analysis) as well as the data, overview plots were generated summarizing the outcome of these *in silico* analyses (Supporting Material S1).

#### Based on mass spectrometry fragmentation spectra identification

An automated pipeline was developed to reprocess the PRIDE (27,28) repository to identify micropeptides. The sequence searching pipeline consisted of pride-asap (29) to extract and infer the correct search parameters, SearchGUI (30) version 2.0.4 for the search engine management and finally PeptideShaker (31) version 1.0.1 for the post-processing of the algorithms output and the filtration for validated spectra.

To minimalize the chances of erroneously assigning a spectrum to a sORF instead of an known human protein, a two stage search approach was used: a filtering search identifying all spectra at a 1% FDR rate at the PSM level against human UniProt-KB (32,33) including isoforms, release 10_2015 and the cRAP library (34) (i), and a follow up search of the non-validated spectra against a sequence database containing the hypothetical sequences of sORF translation products (ii).

The PRIDE ReSpin results are represented on the sORF detail page and can be queried from the BioMart query interface. More information can be found in Supporting Material S2.

### sORFs.org access

**Figure 2.**
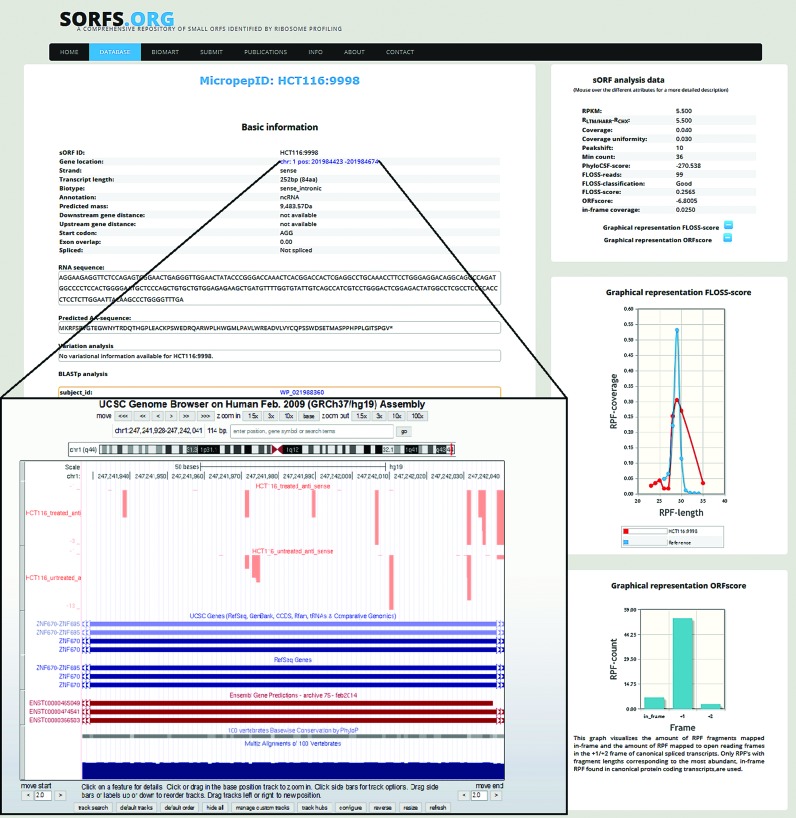
sORF detail page.

**Figure 3.**
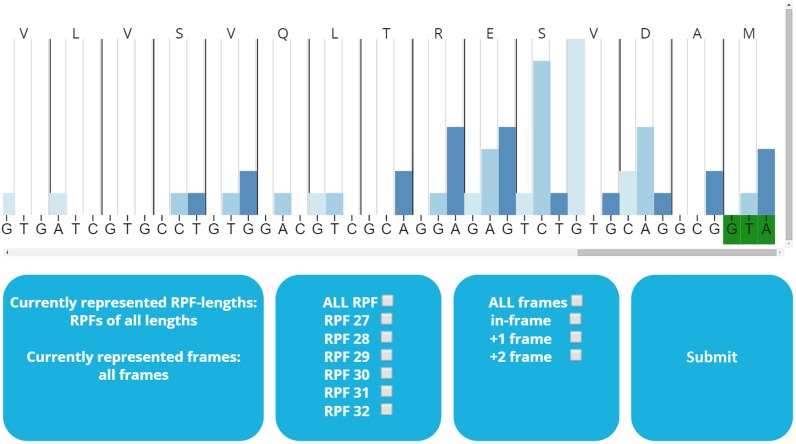
RIBO-SEQ visualization tool with options.

sORFs.org is publicly available through a web interface located at (http://www.sorfs.org). sORFs.org has two different query interfaces, the default query interface (http://www.sorfs.org/database) allows to query on basic sORF attributes (ID, species, cell line, genomic position, length, annotation, biotype, sequence). Additionally a BioMart query interface (http://www.sorfs.org/BioMart) allows to query on all possible features and export the filtered data. A manual is provided for both query interfaces next to the corresponding query interface page. All sORFs can be individually inspected on a detail page (Figure 2), displaying all the sORF attributes. This detail page also contains a RIBO-seq visualization tool, permitting manual inspection of RIBO-seq data. The visualization tool enables selection of RPFs based on length or reading frame (Figure 3). Furthermore the detail page contains a hyperlink through the ‘gene location’ attribute, where the mapped RIBO-seq data are available for inspection in the UCSC browser (35,36). Researches can submit data and papers through the ‘submit’ (http://www.sorfs.org/submit) page and sORFs.org can be contacted through the ‘contact’ (http://www.sorfs.org/contact) page.

## CONCLUSION AND FUTURE DIRECTION

Although the micropeptide research field has grown significantly, it still remains in its infancy. The existence of micropeptides has been long neglected, but refusing to accept their significance could impair our scientific knowledge. Since the advent of RIBO-seq, various tools and metrics have been developed to discover sORFs. sORFs.org aims to perform these tools and metrics, integrate these various data sources, and furthermore use visualization tools and intuitive querying interfaces to enable wet lab researchers to question this pool of information. Consequently the micropeptide research field will become more accessible. This sORFs.org resource can also significantly facilitate other follow-up analyses. A sORFs sequence database can be constructed to use in MS-based identification. Also, certain (disease) phenotype related variations could be explained because they reside within a sORF, encoding a functional micropeptide.

As RIBO-seq becomes more appreciated, sORFs.org is expected to elaborate on the number of data sets and supported species. Simultaneously new tools and metrics will be incorporated following new developments in the field. For instance, a pipeline is being developed to allow sORF identification from RIBO-seq data lacking HARR/LTM treatment. sORFs.org contains the potential to become a community resource for sORFs and micropeptide research.
